# Psychotic relapse and associated factors among patients attending health services in Southwest Ethiopia: a cross-sectional study

**DOI:** 10.1186/s12888-016-1076-2

**Published:** 2016-10-20

**Authors:** Mahlet Fikreyesus, Matiwos Soboka, Garumma Tolu Feyissa

**Affiliations:** 1 Amanuel Mental Health Specialized Hospital, Addis Ababa, Ethiopia; 2 Department of Psychiatry, Jimma University, Jimma, Ethiopia; 3 Department of Health Education and Behavioral Science, Jimma University, Jimma, Ethiopia; 4 The Joanna Briggs Institute, University of Adelaide, Adelaide, Australia

**Keywords:** Relapse, Psychotic disorders, Substance use, Medication side effect, Medication compliance, Ethiopia

## Abstract

**Background:**

Psychotic relapse leads to repeated hospitalization and negatively affects the clinical prognosis of the patients. Information on prevalence of relapse among patients with psychotic disorders in Ethiopian setting is scarce. This study aimed to assess the prevalence of relapse among patients with psychotic disorders attending services in Jimma University Specialized Hospital (JUSH).

**Methods:**

Data were collected using interviewer administered questionnaire. We used medication adherence rating scale (MARS) to assess compliance to medication and abnormal involuntary movement scale (AIMS) to detect medication side effects. Logistic regression analysis was used to identify independent predictors of psychotic relapse. All variables with *P*-value <0.25 in the bivariate analyses were entered into multivariate logistic regression and variables with *P*-value < 0.05 in the final model were declared to be significantly associated with the outcome variable.

**Results:**

The prevalence of relapse among patients with psychotic disorder was 24.6 % (*n* = 95). Of this, 25.4 and 22.4 % were males and females respectively. The odds of developing psychotic relapse among patients living with family was 72 % lower than that of patients living alone (aOR = 0.28, 95 % CI = 0.08, 0.93). The odds of developing psychotic relapse among patients compliant to medication was 69 % lower than that of patients who were not compliant to medications (aOR = 0.31, 95 % CI = 0.12, 0.80). The odds of developing psychotic relapse among patients having high score on social support score was 48 % lower than that of patients who were compliant to medications (aOR = 0.52, 95 % CI = 0.28, 0.95). The odds of developing psychotic relapse among patients reporting to have sought religious support was 45 % lower than that of patients who have not sought religious support (aOR = 0.55, 95 % CI = 0.31, 0.96). On the other hand, the odds of developing psychotic relapse among participants who have experienced medication side effects was 1.83 times higher when compared to those who have never experienced medication side effects (aOR = 1.83, 95 % CI = 1.01, 3.31).

**Conclusions:**

The high prevalence of relapse among patients with psychotic disorder needs special attention. Clinicians need to pay attention to medication side effects the patient faces. Intervening noncompliance to medication and appropriately managing medication side effects may help in preventing psychotic relapse that may result because of non-compliance. The provision of counseling, psycho education, psycho social support may help patients in improving compliance to medication and reducing psychotic relapse. Developing and strengthening community based rehabilitation services should be emphasized as part of mental healthcare services.

## Background

Mental illness is among the prevalent non-communicable diseases worldwide [1]. Mental health negatively affects both the patient and the family members financially and socially [2]. The stigma attached to schizophrenia creates a vicious cycle of discrimination leading to social isolation, unemployment, drug abuse, long lasting institutionalization or even homelessness, which further decreases the chances of recovery. Families of patients carry the burden of caring for patients through restricted social activities and economic problems [3]. Relapse costs a lot for patients and their families and imposes a financial burden on hospital and community services [4]. Costs for relapse cases were four times higher than that of non-relapse cases [5]. Relapse cases are associated with hospital admissions. The cost of hospitalization contributes to one- to two- third of the cost for the care of schizophrenia [6]. Relapses may also lead the patients to stay on medication for a longer period of time [7]. Relapse was associated with medication side effects [8–11], substance use [12–15], long stay before initiating therapy [16–18] and comorbid medical or psychiatric illness [19–21].

A study conducted in rural Ethiopia showed that, families of patients diagnosed with schizophrenia had spent $16.52 out-of-pocket and on average spent 3.12 (SD = 4.54) days of work due to care giving in one month [22]. However, there is limited data on prevalence of relapse and associated factors among patients with psychotic disorders. Therefore, this research aimed at assessing the prevalence of, and risk factors for relapse among patients with psychotic disorders.

## Methods

### Study setting

This cross-sectional study was conducted in Jimma University specialized hospital (JUSH), which is located in southwestern part of Ethiopia 354 km far from Addis Ababa. Jimma University specialized hospital provides psychiatric services for inpatient and outpatient services [23]. The outpatient psychiatry clinic at JUSH was established in 1988. Data collection for this research was conducted in November, 2013.

### Sample size determination and sampling procedure

The sample size for the study was determined by assuming relapse rate of 50 % giving any particular out come to be with in 5 % marginal error and 95 % confidence interval of certainty (alpha = 0.05). Based on this assumption, the actual sample size calculated for this study was 386. All adult patients were invited to participate in the study consecutively.

### Data collection instrument

Data were collected using a structured interviewer administered questionnaire on the following variables:

- **Relapse among patients with psychotic disorder**
  A structured interviewer administered questionnaire containing a yes or no question was used to assess relapse. We assessed relapse based on the DSM IV-TR relapse definition criteria [24]. Patients were asked about the numbers of relapses they had. In addition, we reviewed their charts to identify the recorded numbers of relapses.
- **Factors associated with relapse**
  - *Socio demographic factors*: The structured questionnaire contained questions on socio-demographic and socio-economic status of the patients (age, sex, marital status, and educational level, and ethnicity, frequency of attending a place of worship, occupation, income and living condition).
  - *Medication related factors*: We used medication adherence scale (MARS) to assess adherence to anti-psychotic medication [25]. Patients who responded ‘no’ for questions numbers 1–6 and 9–10 and ‘yes’ for question numbers 7–8 were considered as compliant. And those patients who responded ‘yes’ for question numbers 1–6 and 9–10 and ‘no’ for question numbers 7–8 were considered as non-compliant [26]. Also abnormal involuntary movement scale (AIMS) was used to assess medication side effects. The AIMS is considered as standard clinical practice for patients receiving long term neuroleptic drugs and it was applied by clinicians [27, 28].
  - *Substance use disorders*: We utilized CAGE (cudown, annoyed, guilty and eye opener) to screen alcohol use disorder. Two or more positive responses on CAGE are indicative of alcoholism [29]. We used Drug abuse screening test (DAST) to assess khat abuse [30]. Khat is a leaf, which is one among psychoactive substances and it is commonly used in East Africa including Ethiopia [31, 32] The interpretation of DAST is follows: a score of zero indicates absence of substance abuse, 1–2 indicate low level of substance abuse, 3–5 indicate moderate level of substance abuse, 6–8 indicate substantial level of substance abuse and 9–10 indicate severe level of substance abuse. Those participants having moderate level of substance abuse need referral to professionals [33–35].
  - *Duration of untreated psychotic disorder and Comorbidity*: The participants were asked to indicate the duration of time they stayed before they sought therapy at modern health services after the onset of the illness. In addition, we asked the places they went before they sought services at modern health institutions.
  - *Social support*: We measured social support using six items. Each question had two parts. The first part is about the number of individuals that participants listed as a source of support and the second one is their level of satisfaction [36].

### Data collection procedures

The questionnaire was pretested on a sample equal to 5 % of the total sample size that were not part of the main study. Data were collected by interviewing patients with diagnosis of psychotic disorders coming to JUSH psychiatry clinic for follow-up purposes. Three psychiatric nurses and two postgraduate students attending their studies in masters program in integrated community and clinical mental health (ICCM) collected the data. Two students attending masters program in ICCM and the principal investigator supervised the data collection. Before the data collection, a two-days training was given for the data collectors and suppervisors. The training included detailed information on how to collect data, how to score the different scales used in the study and how to perform AIMS. The supervisor monitored data quality and checked all questionnaires for completeness.

### Data analysis

Data were checked for completeness, and were entered into Epi-Data version 3.1. After double entry verification, the data were exported to SPSS (statistical package for social science) version 20 for analyses.

The dependent variable (relapse) and independent variables (predictors) were entered into bivariate logistic regression in order to determine statitical association between dependent and independent variables. All variables associated with the dependent variable with *p*-value less than 0.25 in the bivariate analyses of the binary logistic regression were entered into multivariate models of the logistic regression by enter method in order to identify interaction between variables and to control for potential confounders. Variables with *p*-value < 0.05 in the multivariate analyses were considered significant predictors of relapse.

## Results

### Paricipants characteristics

A total of 386 participants were approached to participate in the study and all of them agreed to participante with the response rate of 100 %. Nearly 72.3 % (*n* = 279) of participants were males; 61.9 % (*n* = 239) were Oromo in ethnic origin. Of the total participants, nearly 61 % (*n* = 234) were Islamic religion followers followed by Orthodox Christians (25.1 %; *n* = 97). Out of the total participants of this study, 73.5 % (*n* = 280) were diagnosed as cases of schizophrenia. The mean age of the particpants was 31.49 ± SD 9.36 years ranging from 18 to 68 and 48.2 % (*n* = 186) were between the ages of 25–34 years. Nearly, half of the the study participants (48.7 %; *n* = 188) were married. Amongst the total study participants, 29 % (*n* = 112) were unemployed. The mean monthly income of the study participants was 1446.13 ± 1291.27 Ethiopian Birr (1 USD = 20.91 ETB) (see Table 1).

**Table 1:** Socio-demographic characteristics of patients with psychotic disorders, Jimma University Specialized Hospital, Ethiopia, 2013 (*n* = 386)

Socio demographic characteristics	Number (percent)
Sex	
Male	279 (72.3 %)
Female	107 (27.7 %)
Age	
18–24	79 (20.5 %)
25–34	186 (46.2 %)
35–44	82 (21.2 %)
45–54	28 (7.3 %)
55–68	11 (2.8 %)
Religion	
Orthodox	97 (25.1 %)
Muslim	234 (60.4 %)
Protestant	51 (13.2 %)
Catholic	1 (0.3 %)
Other*	3 (0.8 %)
Frequency	
Frequent	170 (44 %)
Sometimes	196 (50.8 %)
Never	20 (5.2 %)
Ethnicity	
Oromo	239 (61.9 %)
Amhara	66 (17.1 %)
Gurage	19 (4.9 %)
Kaffa	26 (6.7 %)
Dawro	20 (5.2 %)
Yem	5 (1.3 %)
Other**	11 (2.8 %)
Marital status	
Single	179 (46.4 %)
Married	188 (48.7 %)
Divorced	11 (2.88 %)
Separated	2 (0.5 %)
Widowed	6 (1.6 %)
Educational	
Illiterate	50 (13 %)
Primary	176 (45.6 %)
Secondary	96 (24.9 %)
Tertiary	64 (16.6 %)
Occupation	
Unemployed	112 (29 %)
gov't employee	87 (22.5 %)
Merchant	26 (6.7 %)
Private	29 (7.5 %)
Farmer	97 (25.1 %)
Other***	35 (9.1 %)
Income	
167–541	96 (24.9 %)
542–1000	120 (31.1 %)
1001–1888	75 (19.4 %)
1889–10000	95 (24.6 %)

Other* includes Jehovah’s witnesses

Other** includes Tigrae, Wolayita and Hadiya

Other*** includes wood workers, daily laborer, house wife and students

### Prevalence of relapse among patients with psychotic disorders

Out of the total participants, 24.6 % (*n* = 95) had at least one relapse or reoccurrence of psychotic symptoms within the past 6 months; 14.8 % (*n* = 57) had only one relapse; 9.8 % (*n* = 38) had more than one relapse. Prevalence of relapse among male participants was 25.4 % (*n* = 71). Nearly one fourth (25.8 %, *n* = 48) of patients with relapse were found among the patients in the age group of 25–34 years (see Table 2).

**Table 2:** Association of relapse with socio demographic characteristics (bivariate logistic regression)

Variables	N	Relapse	No relapse	*P*-value	Crude OR	95 % CI
Sex						
Male	279	71 (25.4 %)	208 (74.6 %)	0.54	1.18	0.50, 2.00
Female	107	24 (22.4 %)	83 (77.6 %)		Reference	
Age						
18–24	79	14 (17.7 %)	65 (82.3 %)	0.16	0.62	0.39, 1.20
25–34	186	48 (25.8 %)	138 (74.2 %)		Reference	
35–44	82	23 (28 %)	59 (72 %)	0.70	1.12	0. 63, 2.01
45–54	28	9 (32.1 %)	19 (67.9 %)	0.48	1.36	0. 58, 3.21
55–68	11	1 (9.1 %)	10 (90.9 %)	0.24	0.29	0. 04, 2.31
Ethnicity						
Oromo	239	55 (23 %)	184 (77 %)		Reference	
Amhara	66	20 (30.3 %)	46 (69.7 %)	0.23	1.46	0. 79, 2.66
Gurage	19	4 (21.1 %)	15 (78.9 %)	0.85	1. 89	0. 28, 2.80
Kaffa	26	4 (15.4 %)	22 (84.6 %)	0.379	0.61	0. 20, 1.84
Dawro	20	7 (35 %)	13 (65 %)	0.23	1.80	0. 69, 4.74
Yem	5	1 (20 %)	4(80 %)	0.87	0.84	0. 09, 7.64
Other*	11	4 (36.4 %)	7 (63.6 %)	0.32	1.91	0. 54, 6.77
Religion						
Orthodox	97	25 (25.8 %)	72 (74.2 %)	0.79	1.08	0. 63, 1.86
Muslim	234	57 (24.4 %)	177 (75.6 %)		Reference	
Other**	55	13 (23.6 %)	42 (76.4 %)	0.91	0.96	0. 48, 1.92
Frequency of worship						
Frequently	170	35 (20.6 %)	135 (79.4 %)		Reference	
Sometime	196	51 (26 %)	145 (74 %)	0.22	1.36	0.83, 2.21
Never	20	9 (45 %)	11 (55 %)	0. 02	3.16	1.21, 8.21
Marital status						
Single	179	44 (24.6 %)	135 (75.4 %)	0.93	0.98	0. 61, 1.57
Married	188	47 (25 %)	141 (75 %)		Reference	
Divorced	11	1 (9.1 %)	10 (90.9 %)	0.26	0.30	0. 04, 2.41
Separated/widowed	8	3 (37.5 %)	5 (62.5 %)	0.43	1.80	0. 41, 7.82
Educational status						
Illiterate	50	12 (24 %)	38 (76 %)	0.65	1.19	0. 56, 2.50
Primary	176	37 (21 %)	139 (79 %)		Reference	
Secondary	96	19 (19.8 %)	77 (80.2 %)	0.81	0.93	0. 50, 1.72
tertiary	64	27 (42.2 %)	37 (57.8 %)	0.001	2.74	1.48, 5.07
Occupation						
Unemployed	112	25 (22.3 %)	87 (77.7 %)		Reference	
Government employee	87	29 (33.3 %)	58 (66.7 %)	0.09	1.74	0. 93, 3.27
Merchant	26	4 (15.4 %)	22 (84.6 %)	0.44	0.63	0.20, 2.01
Farmer	97	23 (23.7 %)	74 (76.3 %)	0.81	1.08	0. 57, 2.06
Other***	64	14 (21.9 %)	50 (78.1 %)	0.95	0.97	0.46, 2.04
Income						
167–541	96	21 (21.9 %)	75 (78.1 %)	0.73	1.12	0.58, 2.17
542–1000	120	24 (20 %)	96 (80 %)		Reference	
1001–1888	75	26 (34.7 %)	49 (65.3 %)	0.02	2.12	1.11, 4.08
1889–10000	95	24 (25.3 %)	71 (74.7 %)	0.36	1.35	0.71, 2.57
Living condition						
Alone	32	5(15.6 %)	27(84.4 %)		Reference	
With family	339	88(26 %)	251(74 %)	0.20	0.53	0.20, 1.41
With relative	15	2(13.3 %)	13(86.7 %)	0.28	0.44	0.10, 1.98

Other* includes Wolayita, Tigrie and Hadiya

Other**includes Protestant, Catholic and Jehovah witness

Other*** includes wood workers, daily laborers, house wives and students

Nearly one-fourth of participants who were diagnosed as having schizophrenia (25 % *n* = 70), schizophreniform and schizoaffective disorders (25.5 %, *n* = 13) had psychotic relapse (Table 3).

**Table 3:** Association of relapse with the type of psychotic diagnosis (bivariate logistic regression)

Variables	N	Relapse	No relapse	*P*-value	Crude OR	95 % CI
Diagnosis						
Schizophrenia	280	70 (25 %)	210 (75 %)	0.62	1.19	0.60, 2.39
Schizophreniform and schizoaffective	51	13 (25.5 %)	38 (74.5 %)	0.66	1.23	0.50, 3.01
Brief psychotic	55	12 (21.8 %)	43 (78.2 %)		Reference	

### Factors associated with relapse

Out of the total particpants, only 7.7 % (*n* = 30) reported that they were non-compliant to their medication. Of these, 50 % (*n* = 15) had relapse. Some of the reasons reported by participants for becoming non-compliant to their medication were forgetting to take medications (8 %, *n* = 30), feeling better (11.7 %, *n* = 45) and worsening of the symptoms (9.3 %, *n* = 36). Out of the total particpants, 75.4 % (*n* = 291) reported that, medication makes them feel tired (see Table 4). Abnormal involuntary movement scale (AIMS) was done for all participants and 41 % (*n* = 157) of the participants had scored above the mean (2.99 ± SD 4.26) score. Out of patients who experienced medication side effects, 29.3 % (*n* = 46) reported having had a relapse (see Table 4).

**Table 4:** Association of psychotic relapse with medication related factors (bivariate logistic regression)

Variables	N	Relapse	No relapse	*P*-value	Crude OR	95 % CI
MARS^a^						
Compliant	356	80 (22.5 %)	276 (77.5 %)	0.001	0.29	0.14, 0.62
Non-compliant	30	15 (50 %)	15 (50 %)		Reference	
AIMS^b^						
Below mean	229	49 (21.4 %)	180 (78.6 %)		Reference	
Above mean	157	46 (29.3 %)	111 (70.7 %)	0.08	1.52	0.96, 2.43

^a^ *MARS* medication adherence rating scale

^b^ *AIMS* abnormal involuntary movment scale

Additionally, nearly half, 49 % (*n* = 173) of the total participants were found to drink alcohol. Of this, 7 % (*n* = 27) had alcohol use disorders. Among patients with alcohol use disorders, 40.7 % (*n* = 11) were identified as having a psychotic relapse. Out of this patients, 81.5 % (*n* = 22) were diagnosed with schizophrenia (see Table 5).

**Table 5:** Association of relapse with substance related factors (bivariate logistic regression)

Variables	N	Relapse	No relapse	*P*-value	Crude OR	95 % CI
CAGE^a^						
No problem problem drinking	359	84 (23.4 %)	275 (76.6 %)		Reference	
Problem drinking	27	11 (40.7 %)	16 (59.3 %)	0.05	2.25	1.01, 5.04
DAST^b^						
No	213	43 (20.2 %)	170 (79.8 %)		Reference	
Low	33	4 (12.1 %)	29 (87.9 %)	0.28	0.55	0.18, 1.63
Moderate	114	36 (31.6 %)	78 (68.4 %)	0.02	1.83	1.09, 3.06
Substantial	26	12 (46.2 %)	14 (53.8 %)	0.004	3.39	1.46, 7.85

^a^Cudown, annoyed, guilty and eye opener

^b^Drug abuse screening test

The prevalence of relapse among patients with low social support was 26.8 % (*n* = 70) (see Table 6). Nearly 29 % (*n* = 61) and 27.7 % (*n* = 41) of participants who sought religious and traditional support before coming to the hospital had relapse respectively (see Table 6). Among patients having comordbid psychiatric illnesses, 30 (33.7 %) of them had psychotic relapse. In the bivariate analyses, having comorbid psychiatric illness was significantly associated with relapse (Table 7).

**Table 6:** Association of relapse with social support, DUP, help seeking (bivariate logistic regression)

Variables	N	Relapse	No relapse	*P*-value	Crude OR	95 % CI
Social support						
Low score	261	70 (26.8 %)	191 (73.2 %)		Reference	
High score	125	25 (20 %)	100 (80 %)	0.15	0.68	0.41, 1.14
DUP^a^						
Below mean	221	50 (22.6 %)	171 (77.4 %)		Reference	
Above mean	165	45 (27.3 %)	120 (72.7 %)	0.30	1.28	0. 81, 2.04
Sought support from traditional sources						
Yes	148	41 (27.7 %)	107 (72.3 %)	0.27	1.31	0.82, 2.09
No	238	54 (22.7 %)	184 (77.3 %)		Reference	
Sought religious support						
Yes	210	61 (29 %)	149 (71 %)		Reference	
No	176	34 (19.3 %)	142 (80.7 %)	0.03	0.59	0.36, 0.94

^a^ *DUP* duration of untreated psychoses

**Table 7:** Association of relapse with comorbidity among the patients (bivariate logistic regression)

Variables	N	Relapse	No relapse	*P*-value	Crude OR	95 % CI
Comorbid psychiatric illness						
Yes	89	30 (33.7 %)	59 (66.3 %)	0.02	1.82	1.08, 3.05
No	297	65 (21.9 %)	232 (78.1 %)		Reference	
Comorbid medical illness						
Yes	24	9 (37.5 %)	15 (62.5 %)	0.14	1.93	0.81, 4.56
No	362	86 (23.8 %)	276 (76.2 %)		Reference	

After adjusting for potential confounders using multivariate logistic regression anlaysis, living with family compared to living alone, being compliant to medication high score social support and seeking religious help were statistically associated with reduced odds of developing psychotic relapse. The odds of developing psychotic relapse among patients living with family was 72 % lower than that of patients living alone (aOR = 0.28, 95 % CI = 0.08, 0.93). The odds of developing psychotic relapse among patients compliant to medication was 69 % lower than that of patients who were not compliant to medications (aOR = 0.31, 95 % CI = 0.12, 0.80). The odds of developing psychotic relapse among patients having high score on social support score was 48 % lower than that of patients who were compliant to medications (aOR = 0.52, 95 % CI = 0.28, 0.95). The odds of developing psychotic relapse among patients reporting to have sought religious support was 45 % lower than that of patients who have not sought religious support (aOR = 0.55, 95 % CI = 0.31, 0.96) (see Table 8).

**Table 8:** Predictors of relapse among patients with psychotic disorders (multivariate logistic regression)

Variables	*P*-value	Adjusted OR	95 % CI	
			Lower	Upper
Sex				
Male	0.75	0.89	0.45	1.76
Female		Reference		
Age				
18–24	0.13	0.53	0 .24	1.20
25–34		Reference		
35–44	0.57	1.22	0.62	2.42
45–54	0.45	1.49	0.53	4.14
55–68	0.25	0.26	0.03	2.63
Ethnicity				
Oromo		Reference		
Amhara	0.81	1.10	0.50	2.45
Gurage	0.43	0.58	0.15	2.22
Kaffa	0.08	0.31	0.08	1.17
Dawro	0.33	1.87	0.54	6.49
Yem	0.76	0.67	0.02	8.58
Other*	0.66	0.65	0.09	4.56
Frequency of worship				
Frequently		Reference		
Sometime	0.18	1.51	0.83	2.77
Never	0.05	3.64	0.99	13.34
Educational status				
Illiterate	0.52	1.34	0.56	3.18
Primary		Reference		
Secondary	0.99	0.99	0.47	2.09
Tertiary	0.01	3.49	1.29	9.47
Occupation				
Unemployed		Reference		
Government employee	0.99	0.99	0.35	2.84
Merchant	0.73	0.79	0.21	2.95
Farmer	0.62	1.25	0.51	3.06
Other***	0.62	1.26	0.51	3.12
Income				
167–541	0.43	1.35	0.64	2.82
542–1000		Reference		
1001–1888	0.10	1.10	0.87	4.58
1889–10000	0.87	1.07	0.49	2.34
Living condition				
Alone		Reference		
With family	0.04	0.28	0.08	0.93
With relative	0.23	0.32	0.05	2.02
MARS^a^				
Compliant	0.02	0.31	0.12	0.80
Non-compliant		Reference		
AIMS^b^				
Below mean		Reference		
Above mean	0.04	1.83	1.01	3.31
CAGE^c^				
No problem of drinking		Reference		
Drinking Problem	0.95	1.04	0.32	3.34
DAST^d^				
No		Reference		
Low	0.48	0.64	0.19	2.19
Moderate	0.16	1.68	0.82	3.45
Substantial	0.11	2.61	0.810	8.42
Social support				
Low score		Reference		
High score	0.04	0.52	0.28	0.95
Religious help seeking				
Yes		Reference		
No	0.04	0.55	0.31	0.96
Comorbid psychiatric illness				
Yes	0.52	0.77	0.35	1.70
No		Reference		
Comorbid medical illness				
Yes	0.22	1.98	0.67	5.90
No		Reference		

^a^ *MARS* ^:^ medication adherence rating scale

^b^ *AIMS* abnormal involuntary movment scale

^c^ *CAGE* cudown, annoyed, guilty and eye opener

^d^ *DAST* drug abuse screening test

Other* includes Wolayita, Tigrie and Hadiya

Other*** includes wood workers, daily laborers, house wives and students

On the other hand, the odds of developing psychotic relapse among participants who have experienced medication side effects was 1.83 times higher when compared to those who have never experienced medication side effects (aOR = 1.83, 95 % CI = 1.01, 3.31) (see Table 8).

## Discussions

In this study, about one-third of the study participants were identified to have relapse. This finding was in agreement with a Canadian study (35.7 %) [37]. However, the finding is lower than study findings from a community cohort study conducted in Butajira Ethiopia, (54 %) [38]. This difference could be due to the difference in the type of the study. While our study was hospital-based, the study conducted in Butajira was a community-based study. This finding may be expected because psychotic patients in the community might not have been on therapy provided in modern health intitutions. In addition, the relapse rate in or study was lower than the study conducted in Hong Kong (48.1 %) [39].

In the current study, lower relapse was associated with living with family member compared to living alone, seeking religious support, high score on social support, and compliance to medication. This was in agreement with the findings of the studies conducted in South Africa and Hong Kong [39, 40]. The odds of having relapse among participants who live with family was 72 % lower than that of patients who live alone. This could be due to the reason that, when patients live alone; they do not have anyone to encourage or remind them to take their medication properly and attend their follow-up regularly. This is in agreement with the study done in Spain, which indicated that cohabitation with other people improves compliance to medication because relatives could serve as supervisors [41].

In this study, the odds of developing psychotic relapse among patients who were compliant to their medication was 69 % lower when compared to the odds among non-compliant patients. This finding is in agreement with the studies conducted in Norway and USA [11, 42]. This underscores the importance of interventions that improve compliance to medication as part of the measures taken to reduce psychotic relapse. Further studies are needed to identify interventions that are effective and feasible in the context for improving compliance to medication.

In the current study the prevalence of relapse among patients with medication side effects was 29.3 % (*n* = 46). An Indian study reported a relapse rate of 37.5 % among patients with medication side effects [43]. This difference could be due to difference in the instrument (AIMS vs. clinical interview). We used AIMS, while the Indian study used clinical interview. The instrument we used for assessing medication side effect (AIMS) assesses only extra pyramidal side effects, and hence, it might have underestimated the magnitude of medication side effects.

In the current study, the odds of developing relapse among patients who have experienced medication side effects was 1.83 times higher when compared to that of patients who have never experienced medication side effects. This underscores the importance of emphasizing detection and treatment of medication side effects among psychotic patients. The use of antipsychotic drugs that are asssociated with less side effects may be considered. A meta-analysis has shown that risperidone use was associated with less side effects compared to haloperidol [44]. In addition, it is essential to link mentally ill persons to NGOs (non-governmental oraganizations) that support recovery and rehablitation of persons with mental disorders. Moreover, developing and strengthening culturally acceptable community rehabilitation intervention and recovery-oriented services that help to develop the skills of patients in dealing with mental health problem is essential. Components such as psycho-education, family intervention, relapse prevention, adherence support, stress and anger management should be included in community based rehabilitation interventions [45].

In the current study, the odds of having relapse among patients who had sought religious support before coming to the hospital was 45 % lower than that of patients who have never sought religious support. This could be because some religious practices help patients to cope with the illness and avoid the use of psychoactive substances [46]. The odds of having psychotic relapse among patients who had high social support score was 48 % lower than that of patients who had low social support score. This finding is in agreement with one meta-analysis, which showed that good social support was associated with less relapse [47, 48]. This indicates the importance of focusing on patients living alone, who have low or no social support. Also, it is essential to arrange referral mechanism for psycho social support services. The psychiatry clinic also needs to employ regular program of psycho education and counseling. In addition, providing support for mentally ill people at the community level is essential. Furthermore, Jimma town health office may consider building rehabilitation centers or providing psycho social support for people who have no social support. The health office also needs collaboration with Jimma town education office to create educational opportunities for mentally ill individuals. To provide support for mentally ill people at the community level, integrating mental health services into urban and rural health extension services may be helpful.

Even though not statistically significant, 40.7 % (*n* = 11) of patients with problem drinking had psychotic relapse. In addition, 31.6 % (*n* = 36) of patients with moderate level of khat abuse had relapse. This could be because of the fact that patients use psychoactive substances to get relief from the symptoms of the illness and side effects. A study conducted in Ethiopia at Ammanuel Hospital on patients with schizophrenia showed that alcohol and khat abuse were contributing factors for relapse of psychotic symptoms and re-hospitalization [15]. Another literature review on substance mis-use and mental illness showed that alcohol use even with small amount was associated with increase symptomatology and rehospitalization [13]. A study conducted in Australia showed that substance mis-use was significantly associated with relapse of positive symptoms [14].

Comorbidity of medical illnesses in this study on patients who had relapse was 37.5 % (*n* = 9). The study conducted in South Africa on patients with schizophrenia showed that 45.5 % (*n* = 6) of patients had comorbid medical illnesses [40].

Nearly 27.3 % (*n* = 45) of participants who had long duration before treatment had relapse of psychotic illness. In this study, duration of untreated psychoses (DUP) was not significantly associated with relapse. Study conducted in USA found that there is positive correlation between DUP and number of relapses [18].

In this study nearly one-forth (*n* = 70) of respondents with diagnosis of schizophrenia had relapse. This could be due to the nature of the illness. Study conducted in Hong Kong on first episode psychosis showed that the diagnosis schizophrenia was associated with increased risk of relapse [39].

Our current study has used standard scales and instruments such as MARS, AIMS, DAST-10, CAGE and SSQ-6. The findings have implications for health program design and service improvement especially for the country and context. Our study has some limitations. First, the instrument we used for assessing medication side effects (AIMS) assesses only extra pyramidal side effects. This might have underestimated the rate of medication side effects faced by participants. Secondly, the study design is cross sectional in nature; hence, it was difficult to identify the cause and effect relationship between study variables.

## Conclusions

In this study, about one-third of the respondents had relapse. Living with family, seeking religious support, high social support score and compliance with medication were independently associated with reduced odds of developing psychotic relapse. Experiencing medication side effects was associated with increased odds of developing psychotic relapse. Clinicians need to detect and manage medication side effects faced by the patients. Intervening noncompliance to medication may help in preventing psychotic relapse that may result because of non-compliance. The linkage of patients to social and religious supports may help the patients in improving compliance to medication and reducing psychotic relapse. Moreover, developing and strenghtneing community based rehabilitation services with components such as psycho education, family intervention, relapse prevention, adherence support and stress and anger management, is needed. Further studies are needed to identify effective and feasible interventions that address compliance to medication.

## Abbreviations

AIMS: Abnormal involuntary movment scale
AOR: Adjusted odds ratio
CAGE: Cudown, annoyed, guilty and eye opener
DALYs: Disability-adjusted life years
DAST: Drug abuse screening test
DSM IV-TR: Diagnostic and statistical manual of mental disorders iv text revision
DUP: Duration of untreated psychoses
JUSH: Jimma University specialized hospital
MARS: Medication adherence rating scale
mhGAP: Mental health gap action program
NGO: Non-governmental organization
OR: Odds ratio
SPSS: Statistical package of social sciences
